# Inheritable and Precise Large Genomic Deletions of Non-Coding RNA Genes in Zebrafish Using TALENs

**DOI:** 10.1371/journal.pone.0076387

**Published:** 2013-10-10

**Authors:** Yun Liu, Daji Luo, Hui Zhao, Zuoyan Zhu, Wei Hu, Christopher H. K. Cheng

**Affiliations:** 1 School of Biomedical Sciences, The Chinese University of Hong Kong, Hong Kong, China; 2 State Key Laboratory of Freshwater Ecology and Biotechnology, Institute of Hydrobiology, Chinese Academy of Sciences, Wuhan, China; 3 Department of Genetics, School of Basic Medical Sciences, Wuhan University, Wuhan, China; 4 School of Biomedical Sciences Core Laboratory, The Chinese University of Hong Kong Shenzhen Research Institute, Shenzhen, China; National University of Singapore, Singapore

## Abstract

Transcription activator-like effector nucleases (TALENs) have so far been applied to disrupt protein-coding genes which constitute only 2–3% of the genome in animals. The majority (70–90%) of the animal genome is actually transcribed as non-coding RNAs (ncRNAs), yet the lack of efficient tools to knockout ncRNA genes hinders studies on their *in vivo* functions. Here we have developed novel strategies using TALENs to achieve precise and inheritable large genomic deletions and knockout of ncRNA genes in zebrafish. We have demonstrated that individual miRNA genes could be disrupted using one pair of TALENs, whereas large microRNA (miRNA) gene clusters and long non-coding RNA (lncRNA) genes could be precisely deleted using two pairs of TALENs. We have generated large genomic deletions of two miRNA clusters (the 1.2 kb *miR-17-92* cluster and the 79.8 kb *miR-430* cluster) and one long non-coding RNA (lncRNA) gene (the 9.0 kb *malat1*), and the deletions are transmitted through the germline. Taken together, our results establish TALENs as a robust tool to engineer large genomic deletions and knockout of ncRNA genes, thus opening up new avenues in the application of TALENs to study the genome *in vivo*.

## Introduction

TALENs are artificial nucleases that consist of the DNA binding domain from transcription activator like effectors (TALE) and a catalytic domain from FokI nuclease [1]–[2]. TALEs bind to DNA through the repeat domain, with one TALE repeat recognizing one DNA base. The base specificity is determined by the 12^th^ and 13^th^ amino acids called repeat-variable di-residues (RVDs) in each TALE repeat, with RVDs NI, NG, HD and NN recognizing adenine (A), thymine (T), cytosine (C) and guanine (G), respectively [3]–[4]. These RVDs-DNA pairings bring the FokI nuclease to a predetermined genomic locus to create DNA double-strand breaks (DSB). Repair of the DSB through the error-prone non-homologous end-joining pathway leads to small indels at the break site, thus enabling targeted gene disruption. So far, TALENs have been employed to disrupt specific genomic loci in yeast [5], worms [1], [6]–[7], plants [8], zebrafish [9]–[13], medaka [14], rat [15], *Xenopus* [16], pig [17] as well as in cell lines [18]–[20] and human stem cells [21].

The indel-mutations generated by TALENs are often small (<30 bp) [22]. So far, these small indel-mutations have been used to disrupt the open reading frames (ORF) of the protein coding genes which constitute only 2–3% of the animal genome. The majority (70–90%) of the animal genome is actually transcribed, producing thousands of ncRNAs such as miRNAs, lncRNA, small interfering RNAs and PIWI-interacting RNAs [23]–[24]. Different from the protein coding genes, the ncRNA genes contain no ORFs [25]. The functional regions of some ncRNAs such as lncRNAs are often unknown [25]. Other ncRNAs such as miRNAs often occur as gene clusters. Therefore, small sequence alterations may not be sufficient to disrupt the functions of these ncRNAs. Increasing evidence have demonstrated the functional roles of ncRNAs in a wide range of biological processes [26]–[28], yet the lack of efficient tools to knockout ncRNA genes in most animal species hinders studies on their *in vivo* functions.

Zebrafish is an important animal model to investigate gene functions. Besides the protein-coding genes, many ncRNA genes in the zebrafish genome are also transcribed [29]–[33]. In this study, we have developed strategies using TALENs to achieve precise and inheritable large genomic deletions and knockout of ncRNA genes in zebrafish.

## Materials and Methods

### Zebrafish Husbandry

AB zebrafish used in this study were maintained at 28°C in the zebrafish facility of the Chinese University of Hong Kong and the Institute of Hydrobiology. All animal experiments were conducted in accordance with the guidelines and approval of the respective Animal Research and Ethics Committees of the Chinese University of Hong Kong and the Institute of Hydrobiology.

### Construction of Customized TALENs

The pCS2-TALEN-ELD/KKR plasmids were constructed as described [16]. Using the modified TALENs vectors, highly effective customized TALENs recognizing 12–31 bp half-sites could be assembled in five days. The whole procedure involves two digestion-ligation steps. The protocol to assemble TALENs was modified from a previous study [34]. The modular plasmids (60 ng each) were digested and ligated in a 10 ul volume containing 1 ul BsaI buffer (NEB buffer 4), 0.6 ul BsaI (6 U, NEB), 0.6 ul T4 ligase (1200 U, NEB) and 0.4 ul of 25 mM ATP. The reaction was performed on a PCR machine for 6 cycles of 20 min at 37°C and 10 min at 16°C, followed by heating to 50°C for 5 min and then to 80°C for 5 min. Thereafter, 1 ul Plasmid Safe DNase (10 U, Epicentre) was added and digested for 30 min. Five ul of the final products were used to transform competent cells. Five white clones were analyzed. The assembled array plasmids were isolated from the correct clones. The second digestion and ligation step was performed in 10 ul volumes containing 60 ng of each array plasmid, pCS2-TALEN-KKR or pCS2-TALEN-ELD, the last repeat plasmid, 1 ul of Esp3I buffer (NEB buffer 3), 0.6 ul of Esp3I (6 U, NEB), 0.4 ul of T4 ligase (800 U, NEB), and 0.4 ul of 25 mM ATP. The reaction was performed on a PCR machine for 6 cycles of 20 min at 37C and 10 min at 16°C, followed by heating to 50°C for 5 min and then to 80°C for 5 min. Five ul of the final products were used to transform competent cells. Plasmids were isolated from the correct clones for DNA sequencing. The zebrafish *nanos*-3′UTR was cloned into the 3′ end of the pCS2-TALEN-ELD/KKR coding sequence between XbaI and NotI. These vectors would be provided upon request.

### TALEN mRNA Preparation, Microinjection and Mutation Detection

To prepare capped TALEN mRNA, the TALEN expression vectors were linearized by NotI and transcribed using the Sp6 mMESSAGE mMACHINE Kit (Ambion). TALEN mRNAs (100–500 pg) were microinjected into one-cell stage zebrafish embryos. The number of normal and deformed embryos were recorded at 24 hours post fertilization (day 1) and 48 hours post fertilization (day 2) (Table S1 in File S1). Two days after injection, genomic DNA was isolated from 8–10 pooled larvae with normal morphology. The target genomic region was amplified by limited cycles of PCR and subcloned to into pMD18-T (Takara) [35]. The mutation was analyzed by PCR or by sequencing. The primers used in this study are listed in Table S2 in File S1.

### Mutation and Deletion Frequency Analysis

To determine the mutation frequency of each targeted locus and the fragment deletion frequency induced by the TALEN pair, genomic DNA was isolated from three replicates of 8–10 pooled zebrafish embryos at 48 hours post fertilization. For mutation frequency analysis, each target locus was amplified and subcloned into pMD18-T. Thirty-two single colonies were analyzed for each sample by PCR or subsequent sequencing. For genomic fragment deletion frequency analysis, real-time PCR was performed on an ABI PRISM 7900 Sequence Detection System (Applied Biosystems) using the SYBR Green I Kit. Standard curves were generated by serial dilution of the plasmid DNA. The genomic fragment deletion frequency was calculated as the copy number of genomic DNA with deletions divided by that of the total genomic DNA.

### Suppression of Target GFP Expression by Wild-type or Mutated miR-1-2

The GFP sensor plasmid containing three imperfect complementary sites to miR-1-2 and the RFP indicator plasmid expressing the wild-type miR-1-2 precursor were from a previous study [35]. The miR-1-2 precursor mutants were generated by mutation PCR and cloned into the 3′ end of the pSP64T+dsRed coding sequence between EcoRI and XhoI. The 3′UTR sequence of *cnn2* was cloned into pCS2-GFP-F vector between the XbaI and XhoI. GFP sensor (100 pg) was coinjected with the wild-type or mutated miR-1-2 (300 pg) into one-cell stage embryos. The embryos were photographed at 24 hours post fertilization.

### Screening of Founders

The TALEN injected embryos were raised to adulthood and outcrossed with wild-type fish. For *miR-1-1*, genomic DNA from 32 pooled F1 embryos of each founder was amplified and subcloned. Thirty-two single clones were analyzed by PCR and sequencing. For genomic fragment deletions, 24 or 32 F1 embryos were collected from each founder and genomic PCR was performed to detect fragment deletion from each single embryo. The fragment deletions were subsequently confirmed by sequencing.

## Results

### Targeted Disruption of Individual miRNA Genes using a Single Talen Approach

MiRNAs are small non-coding RNAs regulating their targets by post-transcriptional mechanisms [26]. Loss-of-function of genes involved in the miRNA biogenesis pathway has revealed wide range biological functions of these tiny RNAs [26], [37]. Here we investigate whether TALENs could be used to knockout miRNAs in zebrafish.

**Figure 1:**
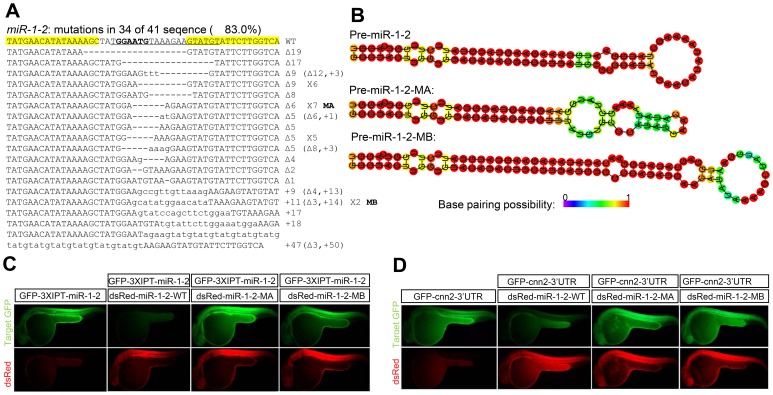
Targeted disruption of zebrafish *miR-1-2*. (**A**) Frequency and spectrum of TALEN induced *miR-1-2* mutations. The TALEN binding sites are shown in yellow background. DNA sequence encoding the mature miR-1-2 is underlined, with the seed sequence in bold. Deletions are indicated by dash lines and insertions are indicated by lowercase letters. The sizes of the insertions (+) or deletions (Δ) and the number of times each mutant allele appearing are shown on the right side of the mutant allele. (**B**) The hairpin structure of the wild-type pre-miR-1-2 and two pre-miR-1-2 mutants (MA and MB) in Panel A. (**C**–**D**) Functional suppression of target GFP expression by the wild-type or two mutated pre-miR-1-2. Messager RNA of GFP sensor (GFP-3XIPT-miR-1-2 or GFP-*cnn2*-3′UTR) and RFP indicator expressing wild-type or mutant pre-miR-1-2 was co-injected into one-cell stage zebrafish embryos. Pictures were taken at 24 hours after injection. GFP-3XIPT-miR-1-2, GFP sensor containing three imperfect complementary sites to miR-1-2; GFP-*cnn2*–3′UTR, GFP sensor containing the 3′UTR sequence of *cnn2* (a miR-1-2 target gene); dsRed-miR-1-2, RFP indicator expressing mature miR-1-2.

Using our optimized TALEN platform [16], we have assembled TALENs for two zebrafish miRNAs (*miR-1-1* and *miR-1-2*). We placed the miRNA seed (a critical region for miRNA-mRNA pairing) at the spacer region where indels often occur. The assembled TALENs induced somatic mutations with high frequencies of up to 97% (Fig. 1A and Fig. S1). Nearly all these mutations altered the miRNA seed sequences, thus leading to loss-of-function of the miRNAs. Moreover, the indels also altered the hairpin structures of the pre-miRNAs (Fig. 1B), conceivably leading to aberrant miRNA biogenesis. Consistent with these predictions, functional studies indicated that the wild-type pre-miR-1-2 but not the pre-miR-1-2 mutants could effectively suppress the target GFP reporters containing either three imperfect complementary sites to *miR-1-2* or the 3′UTR sequence of a reported *miR-1-2* target gene [36] (Fig. 1C and Fig. 1D).

### Precise Large Genomic Deletions of miRNA Clusters using a Dual Talen Approach

**Figure 2:**
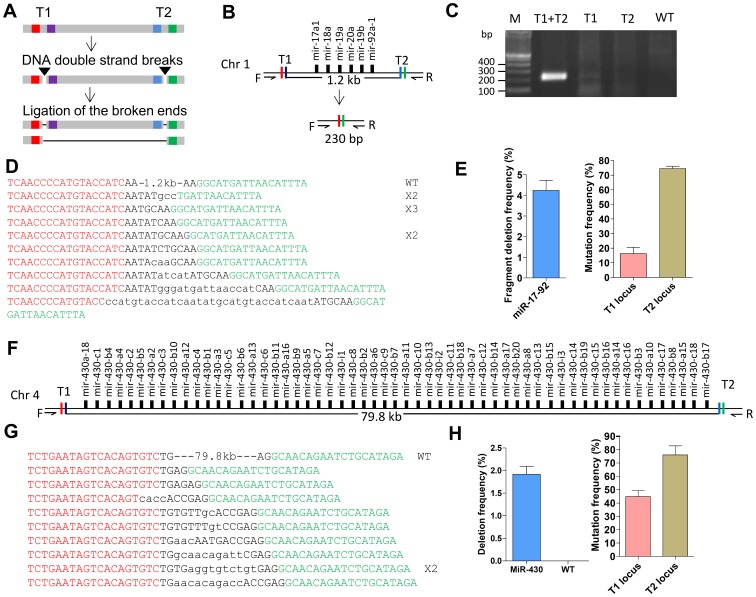
Precise large genomic deletions of miRNA gene clusters. (**A**) Schematic diagram of the large genomic deletion strategy using dual TALEN. The TALEN binding sites are shown in color. T1, TALEN pair 1; T2, TALEN pair 2. (**B**) Schematic representation of the zebrafish *miR-17-92* cluster and the designed TALENs for its deletion. After genomic deletions, a band of about 230 bp is expected to be amplified by PCR using the designed primers. (**C**) Genomic PCR showing deletion of the *miR-17-92* cluster. Gel picture showing PCR amplification of genomic DNA isolated from the pooled zebrafish embryos microinjected with two pairs of TALENs, one pair of TALENs or the wild-type control. (**D**) Sequencing results confirmed deletion of the *miR-17-92* cluster. Alien nucleotides inserted are indicated by lowercase letters. The number of times each mutant allele appearing are shown on the right side of the mutant allele. (**E**) Deletion frequency of the *miR-17-92* cluster and indel-mutation frequency of the T1 and T2 loci. Genomic DNA was isolated from pooled zebrafish embryos injected with the two pairs of TALENs. Data shown are mean values ± S.E.M from three replicates. (**F**) Schematic representation of the zebrafish *miR-430* cluster and the designed TALENs. (**G**) Sequencing results confirmed deletion of the *miR-430* cluster. Alien nucleotides inserted are indicated by lowercase letters. (**H**) Deletion frequency of the *miR-430* cluster and indel-mutation frequency of the T1 and T2 loci. Genomic DNA was isolated from pooled zebrafish embryos injected with the two pairs of TALENs. Data shown are mean values ± S.E.M from three replicates.

More than half of the miRNA genes occur as gene clusters in many vertebrates genome [38]. We thereafter asked whether TALENs could be used to knockout miRNA gene clusters. To knockout gene clusters of large genomic regions, we have devised a strategy that creates two DSBs simultaneously on each side of the targeted genomic fragment using two pairs of TALENs. Repair of the DSB by ligation of the broken ends would lead to disruption of each targeted locus and deletion of the flanked genomic fragment (Fig. 2A). To test this dual TALEN strategy we designed two pairs of TALENs to delete the zebrafish *miR-17-92* cluster (1.2 kb) and the *miR-430* cluster (79.8 kb) (Fig. 2B and Fig. 2F). While the *miR-17-92* cluster encodes 6 miRNAs, the *miR-430* cluster is the largest miRNA cluster in vertebrates encoding 57 miRNAs. To create two concurrent DSBs on each targeted genomic region, mRNA of two pairs of TALENs were co-injected into one-cell stage zebrafish embryos. Two days after injection, genomic DNA was isolated from 8–10 pooled embryos. Primers were designed to detect deletion of the targeted genomic fragment (Table S2, Fig. S2 and Fig. S3 in File S1). PCR amplification of genomic DNA isolated from the pooled embryos indicated successful deletion of these miRNA clusters (Fig. 2C and Fig. S3 in File S1). Sequencing of the PCR products confirmed such successful deletions (Fig. 2D and Fig. 2G). All these deletions occurred accurately between the two targeted sites and there were none or few alien nucleotides retained after the large genomic deletions. Taken together, these results indicate that this dual TALEN strategy provides a powerful approach to generate precise large deletions in the genome.

The fragment deletion frequency of each targeted miRNA gene cluster and the indel-mutation frequency of each targeted locus produced by the dual TALEN approach were systematically analyzed. To evaluate the fragment deletion frequency, two pairs of primers were used to amplify genomic DNA with fragment deletions and a nearby undisrupted genomic region (Table S2 in File S1). Quantitative real-time PCR was performed to calculate the copy number of genomic DNA with fragment deletions with respect to that of the undisrupted genomic DNA. The mean deletion frequency was 4.2% for the *miR-17-92* cluster and 1.9% for the *miR-430* cluster in zebrafish embryonic cells respectively (Fig. 2E and Fig. 2H). The dual TALEN treatment produced indel mutations on each targeted locus with high frequency (Fig. 2E, Fig. H and Table S3 in File S1). For both gene clusters, the fragment deletion frequency is lower than that of the indel mutations.

### Precise Large Genomic Deletions of lncRNA Gene using the Dual talen Approach

**Figure 3:**
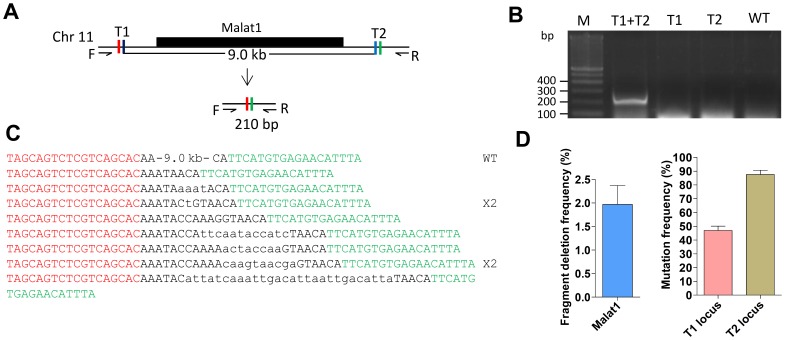
Precise large genomic deletion of a lncRNA gene. (**A**) Schematic representation of the zebrafish lncRNA *malat1* and the TALENs designed for its deletion. After the genomic deletion, a band of about 210 bp is expected to be amplified by PCR using the designed primers. (**B**) Genomic PCR showing the deletion of *malat1*. Gel picture showing PCR amplification of genomic DNA isolated from the pooled zebrafish embryos microinjected with two pairs of TALENs, one pair of TALENs or the wild-type control. (**C**) Sequencing results confirming *malat1* deletions. Alien nucleotides inserted are indicated by lowercase letters. The number of times each mutant allele appearing are shown on the right side of the mutant allele. (**D**) Deletion frequency of the *malat1* and mutation frequency of the two targeted loci. Genomic DNA was isolated from pooled zebrafish embryos injected with the two pairs of TALENs. Data shown are mean values ± S.E.M from three replicates.

Apart from small RNAs, the animal genome also produces large RNA transcripts called lncRNAs longer than 200 nucleotides that do not code for proteins [27], [39]. In zebrafish, two recent studies annotated more than 1000 lncRNA genes that are expressed during early development [32]–[33]. To determine whether this dual TALEN strategy is also applicable to delete lncRNA genes, we have designed two pairs of TALENs to delete the 9.0 kb genomic region encoding the lncRNA *malat1* (Fig. 3A and Fig. 3B). Using this approach we obtained deletion of *malat1* in zebrafish somatic cells with a mean deletion frequency of 2.0% (Fig. 3D and Fig. S4 in File S1). The genomic sequence between the two TALEN targeting sites was accurately deleted (Fig. 3C). These results indicate that the dual TALEN strategy is a powerful approach to knockout lncRNA genes.

### Germline Transmission of Knockout Genotypes

**Figure 4:**
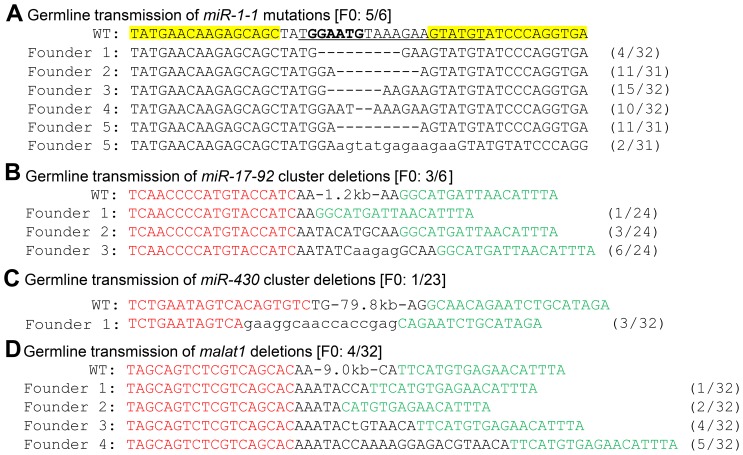
Germline transmission of the knockout genotypes. (**A**) Germline transmission of the *miR-1-1* mutations. The TALEN binding sites are shown in yellow background. DNA sequence encoding the mature miR-1-1 is underlined, with the seed sequence in bold. Deletions are indicated by dash lines and insertions are indicated by lowercase letters. The F0 germline deletion ratio is shown in square brackets. The ratios of mutated/total analyzed sequences from the pooled F1 embryos of each founder are shown in brackets. (**B**–**D**) Germline transmission of the *miR-17-92* deletions (**B**), *miR-430* deletions (**C**) and *malat1* deletions (**D**). The F0 germline deletion ratios are shown in square brackets and the F1 inheritance ratios are shown in brackets.

To test whether these knockout genotypes were inheritable, we have outcrossed the P0 fish raised from the TALEN injected embryos. For *miR-1-1*, 5 out of the 6 P0 fish transmitted the *miR-1-1* indel mutations with high frequencies (Fig. 4A). For the *miR-17-92* cluster, we have genotyped 24 individual F1 embryos from each of the 6 outcrossed P0 fish. Three out of the 6 P0 fish transmitted the 1.2 kb *miR-17-92* deletions to 1/24, 3/24 and 6/24 F1 embryos respectively (Fig. 4B). For *malat1*, 4 out of the 32 P0 fish transmitted the 9.0 kb *malat1* deletions to 1/32, 2/32, 4/32 and 5/32 F1 embryos respectively (Fig. 4D).

For the larger *miR-430* cluster deletions, we have outcrossed 32 P0 fish but failed to obtain germline deletions, suggesting that large genomic deletions are not efficiently transmitted. To overcome this limitation, we have incorporated the zebrafish *nanos*-3′UTR into the TALEN construct (Fig. S5 in File S1). By screening 23 P0 fish raised from the TALEN-*nanos*-3′UTR injected embryos, we have obtained 1 founder transmitted the *miR-430* deletion to 3/32 embryos (Fig. 4C). These data suggested that the *nanos*-3′UTR may increase the germline transmission frequency of large genomic deletions.

## Discussion

Recently the ENCODE project estimated more than 70% of the human genome is transcribed into ncRNAs [40]–[41], indicating that ncRNAs represent a substantial portion of the transcriptome. However, because of the lack of an efficient ncRNA gene knockout platform in vertebrates, only a few of the ncRNA genes have been inactivated in mouse. The ability of engineered nucleases to target specific genomic loci across species makes them an attractive option to address this issue. However, the engineered nucleases have been mainly used to induce small indel mutations. Because the functional regions of ncRNA gene are often unknown, knockout of an entire ncRNA gene is preferred to eliminate gene function. Here we test whether TALENs could be used to knockout of ncRNA genes in zebrafish.

Using zinc finger nucleases and TALENs, targeted fragment deletions have been recently reported in cell lines [17], [42]–[44]. More recently, genomic deletions of an 800 bp fragment using TALENs have been reported in silkworm [7]. During the time this manuscript was reviewed, others have also reported that large genomic deletions could be efficiently engineered in zebrafish using a dual TALEN strategy [45]–[47]. In this study, we have demonstrated that the dual TALEN approach is a robust means to generate inheritable large genomic deletions in zebrafish. First, large genomic deletions (1–80 kb) could be efficiently generated in a few days and the deletions could be readily detected by genomic PCR. Second, all deletions have accurately occurred and none or few alien nucleotides are retained after the genomic deletions (Fig. S6 in File S1). Third, although the fragment deletion frequency is lower than that of the indel-mutation, these large deletions of about 9 kb could be efficiently transmitted through the germline.

Analysis of the transcriptome revealed that many small regulatory RNA are transcribed from the eukaryotic genome [40], [48]. miRNA are small RNA molecules regulating the decay and translation of their target mRNAs. Mature miRNAs are generated from cleavage of the hairpin structure of pre-miRNAs by the RNAse III enzymes dicer [49]. In zebrafish, loss-of-function mutations of dicer in the maternal and zygotic embryos lead to multiple developmental defects followed by death on day 5 post-fertilization [50], indicating that miRNAs as a whole play essential roles in embryogenesis. But the exact functional roles of individual miRNAs or miRNAs clusters remain elusive. Moreover, most miRNAs expressed in zebrafish adult tissues may also regulate other physiological processes. In this study, we have demonstrated that large miRNA gene clusters could be precisely deleted using two pairs of TALENs, whereas individual miRNA genes could be inactivated by destruction of the miRNA hairpin structure and miRNA seed using one pair of TALENs, establishing TALEN as a robust method to knockout miRNA genes. Further studies will be performed to analyze the phenotypic changes resulting from deleting of individual miRNA and miRNA clusters.

Apart from small RNAs, the animal genome also produces many lncRNAs [27], [39]. An increasing number of reports revealed that lncRNAs regulate many cellular processes ranging from maintaining embryonic stem cell pluripotency to epigenetic silencing of large genomic regions [28], [39]. In zebrafish, more than 1000 lncRNA genes are expressed during early development [32]–[33]. Moreover, morpholino knockdown of lncRNAs lead to abnormal brain morphogenesis [33]. However, most lncRNAs are not well conserved and their functional regions are mostly unknown, thus limiting the applicability of the morpholino knockdown approach to study these lncRNAs. The dual TALEN strategy described in this study provides a powerful tool to knockout these lncRNA genes.

Germline transmission of the knockout genotypes is critical to obtain the homozygous gene knockout animals. In this study, we have demonstrated that deletions as long as 9.0 kb could be efficiently transmitted through the germline. To improve the germline integration efficiency of larger genomic deletions, we have incorporated the zebrafish *nanos*-3′UTR into the TALEN construct. The *nanos* 3′UTR was reported to protect mRNA from degradation in the primordial germ cells [51], [52], thus may improve the germ cell target efficiency. Using this TALEN-*nanos*-3′UTR construct, we have demonstrated that large genomic deletions of the miR-430 cluster could be transmitted through the germline. This strategy might also be applicable to engineer large genomic deletions in other species.

**Figure 5:**
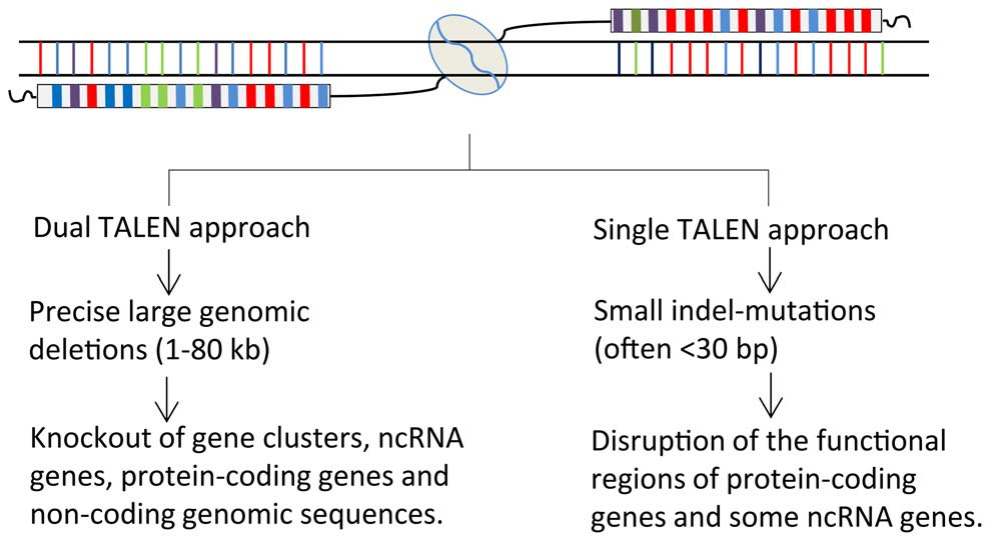
Schematic diagram illustrating knockout strategies using dual or single TALEN approaches. The dual TALEN approach could generate precise large genomic deletions (1–80 kb), allowing knockout of large gene clusters, ncRNA genes, protein-coding genes and non-coding regulatory sequences. The single TALEN approach could produce small indel-mutations of the targeted genomic loci (often <30 bp), allowing disruption of individual protein-coding genes or some individual ncRNA genes.

In summary, we have generated high frequency indel mutations of individual miRNA genes and precise large genomic deletions of non-coding RNA gene clusters in zebrafish using TALENs. Moreover, these deletions are inheritable. We propose that the same TALEN approach could be employed as a molecular tool to study the *in vivo* functions of ncRNA genes in other species. Moreover, the two TALEN strategies described in this study also allow one to knockout other ncRNA genes, entire protein coding genes, genomic regulatory sequences and to disrupt a small functional genomic region depending on the needs (Fig. 5).

## Supporting Information

File S1 **Supporting Figures and Tables.**
(PDF)

## References

[1] Christian M, Cermak T, Doyle EL, Schmidt C, Zhang F, et al (2010) Targeting DNA double-strand breaks with TAL effector nucleases. Genetics 186: 757–761. 20660643 10.1534/genetics.110.120717 PMC2942870

[2] Li T, Huang S, Jiang WZ, Wright D, Spalding MH, et al (2011) TAL nucleases (TALNs): hybrid proteins composed of TAL effectors and FokI DNA-cleavage domain. Nucleic Acids Res 39: 359–372. 20699274 10.1093/nar/gkq704 PMC3017587

[3] Boch J, Scholze H, Schornack S, Landgraf A, Hahn S, et al (2009) Breaking the code of DNA binding specificity of TAL-type III effectors. Science 326: 1509–1512. 19933107 10.1126/science.1178811

[4] Moscou MJ, Bogdanove AJ (2009) A simple cipher governs DNA recognition by TAL effectors. Science 326: 1501. 19933106 10.1126/science.1178817

[5] Li T, Huang S, Zhao X, Wright DA, Carpenter S, et al (2011) Modularly assembled designer TAL effector nucleases for targeted gene knockout and gene replacement in eukaryotes. Nucleic Acids Res 39: 6315–6325. 21459844 10.1093/nar/gkr188 PMC3152341

[6] Wood AJ, Lo TW, Zeitler B, Pickle CS, Ralston EJ, et al (2011) Targeted genome editing across species using ZFNs and TALENs. Science 333: 307. 21700836 10.1126/science.1207773 PMC3489282

[7] Ma S, Zhang S, Wang F, Liu Y, Liu Y, et al (2012) Highly Efficient and Specific Genome Editing in Silkworm Using Custom TALENs. PLoS One 7: e45035. 23028749 10.1371/journal.pone.0045035 PMC3445556

[8] Li T, Liu B, Spalding MH, Weeks DP, Yang B (2012) High-efficiency TALEN-based gene editing produces disease-resistant rice. Nat Biotechnol 30: 390–392. 22565958 10.1038/nbt.2199

[9] Huang P, Xiao A, Zhou M, Zhu Z, Lin S, et al (2011) Heritable gene targeting in zebrafish using customized TALENs. Nat Biotechnol 29: 699–700. 21822242 10.1038/nbt.1939

[10] Sander JD, Cade L, Khayter C, Reyon D, Peterson RT, et al (2011) Targeted gene disruption in somatic zebrafish cells using engineered TALENs. Nat Biotechnol 29: 697–698. 21822241 10.1038/nbt.1934 PMC3154023

[11] Dahlem TJ, Hoshijima K, Jurynec MJ, Gunther D, Starker CG, et al (2012) Simple methods for generating and detecting locus-specific mutations induced with TALENs in the zebrafish genome. PLoS Genet 8: e1002861. 22916025 10.1371/journal.pgen.1002861 PMC3420959

[12] Cade L, Reyon D, Hwang WY, Tsai SQ, Patel S, et al (2012) Highly efficient generation of heritable zebrafish gene mutations using homo- and heterodimeric TALENs. Nucleic Acids Res 40: 8001–8010. 22684503 10.1093/nar/gks518 PMC3439908

[13] Moore FE, Reyon D, Sander JD, Martinez SA, Blackburn JS, et al (2012) Improved somatic mutagenesis in zebrafish using transcription activator-like effector nucleases (TALENs). PLoS One 7: e37877. 22655075 10.1371/journal.pone.0037877 PMC3360007

[14] Ansai S, Sakuma T, Yamamoto T, Ariga H, Uemura N, et al.. (2013) Efficient Targeted Mutagenesis in Medaka Using Custom-Designed Transcription Activator-Like Effector Nucleases (TALENs). Genetics. In press. 10.1534/genetics.112.147645 PMC3583995 23288935

[15] Tesson L, Usal C, Menoret S, Leung E, Niles BJ, et al (2011) Knockout rats generated by embryo microinjection of TALENs. Nat Biotechnol 29: 695–696. 21822240 10.1038/nbt.1940

[16] Lei Y, Guo X, Liu Y, Cao Y, Deng Y, et al (2012) Efficient targeted gene disruption in Xenopus embryos using engineered transcription activator-like effector nucleases (TALENs). Proc Natl Acad Sci U S A 109: 17484–17489. 23045671 10.1073/pnas.1215421109 PMC3491516

[17] Carlson DF, Tan W, Lillico SG, Stverakova D, Proudfoot C, et al (2012) Efficient TALEN-mediated gene knockout in livestock. Proc Natl Acad Sci U S A 109: 17382–17387. 23027955 10.1073/pnas.1211446109 PMC3491456

[18] Mussolino C, Morbitzer R, Lutge F, Dannemann N, Lahaye T, et al (2011) A novel TALE nuclease scaffold enables high genome editing activity in combination with low toxicity. Nucleic Acids Res 39: 9283–9293. 21813459 10.1093/nar/gkr597 PMC3241638

[19] Miller JC, Tan S, Qiao G, Barlow KA, Wang J, et al (2011) A TALE nuclease architecture for efficient genome editing. Nat Biotechnol 29: 143–148. 21179091 10.1038/nbt.1755

[20] Reyon D, Tsai SQ, Khayter C, Foden JA, Sander JD, et al (2012) FLASH assembly of TALENs for high-throughput genome editing. Nat Biotechnol 30: 460–465. 22484455 10.1038/nbt.2170 PMC3558947

[21] Hockemeyer D, Wang H, Kiani S, Lai CS, Gao Q, et al (2011) Genetic engineering of human pluripotent cells using TALE nucleases. Nat Biotechnol 29: 731–734. 21738127 10.1038/nbt.1927 PMC3152587

[22] Hwang WY, Fu Y, Reyon D, Maeder ML, Tsai SQ, et al (2013) Efficient genome editing in zebrafish using a CRISPR-Cas system. Nat Biotechnol 31: 227–229. 23360964 10.1038/nbt.2501 PMC3686313

[23] Kapranov P, Willingham AT, Gingeras TR (2007) Genome-wide transcription and the implications for genomic organization. Nat Rev Genet 8: 413–423. 17486121 10.1038/nrg2083

[24] Amaral PP, Dinger ME, Mercer TR, Mattick JS (2008) The eukaryotic genome as an RNA machine. Science 319: 1787–1789. 18369136 10.1126/science.1155472

[25] Gutschner T, Baas M, Diederichs S (2011) Noncoding RNA gene silencing through genomic integration of RNA destabilizing elements using zinc finger nucleases. Genome Res 21: 1944–1954. 21844124 10.1101/gr.122358.111 PMC3205578

[26] Bartel DP (2009) MicroRNAs: target recognition and regulatory functions. Cell 136: 215–233. 19167326 10.1016/j.cell.2009.01.002 PMC3794896

[27] Mercer TR, Dinger ME, Mattick JS (2009) Long non-coding RNAs: insights into functions. Nat Rev Genet 10: 155–159. 19188922 10.1038/nrg2521

[28] Huarte M (2012) LncRNAs have a say in protein translation. Cell Res. In press. Doi: 10.1038/cr.2012.169. 10.1038/cr.2012.169 PMC3616431 23208421

[29] Wienholds E, Kloosterman WP, Miska E, Alvarez-Saavedra E, Berezikov E, et al (2005) MicroRNA expression in zebrafish embryonic development. Science 309: 310–311. 15919954 10.1126/science.1114519

[30] Chen PY, Manninga H, Slanchev K, Chien M, Russo JJ, et al (2005) The developmental miRNA profiles of zebrafish as determined by small RNA cloning. Genes Dev 19: 1288–1293. 15937218 10.1101/gad.1310605 PMC1142552

[31] Wei C, Salichos L, Wittgrove CM, Rokas A, Patton JG (2012) Transcriptome-wide analysis of small RNA expression in early zebrafish development. RNA 18: 915–929. 22408181 10.1261/rna.029090.111 PMC3334700

[32] Pauli A, Valen E, Lin MF, Garber M, Vastenhouw NL, et al (2012) Systematic identification of long noncoding RNAs expressed during zebrafish embryogenesis. Genome Res 22: 577–591. 22110045 10.1101/gr.133009.111 PMC3290793

[33] Ulitsky I, Shkumatava A, Jan CH, Sive H, Bartel DP (2011) Conserved function of lincRNAs in vertebrate embryonic development despite rapid sequence evolution. Cell 147: 1537–1550. 22196729 10.1016/j.cell.2011.11.055 PMC3376356

[34] Cermak T, Doyle EL, Christian M, Wang L, Zhang Y, et al (2011) Efficient design and assembly of custom TALEN and other TAL effector-based constructs for DNA targeting. Nucleic Acids Res 39: e82. 21493687 10.1093/nar/gkr218 PMC3130291

[35] Sanjana NE, Cong L, Zhou Y, Cunniff MM, Feng G, et al (2012) A transcription activator-like effector toolbox for genome engineering. Nat Protoc 7: 171–192. 22222791 10.1038/nprot.2011.431 PMC3684555

[36] Mishima Y, Abreu-Goodger C, Staton AA, Stahlhut C, Shou C, et al (2009) Zebrafish miR-1 and miR-133 shape muscle gene expression and regulate sarcomeric actin organization. Genes Dev 23: 619–632. 19240126 10.1101/gad.1760209 PMC2658521

[37] Giraldez AJ, Cinalli RM, Glasner ME, Enright AJ, Thomson JM, et al (2005) MicroRNAs regulate brain morphogenesis in zebrafish. Science 308: 833–838. 15774722 10.1126/science.1109020

[38] Thatcher EJ, Bond J, Paydar I, Patton JG (2008) Genomic organization of zebrafish microRNAs. BMC Genomics 9: 253. 18510755 10.1186/1471-2164-9-253 PMC2427041

[39] Rinn JL, Chang HY (2012) Genome regulation by long noncoding RNAs. Annu Rev Biochem 81: 145–166. 22663078 10.1146/annurev-biochem-051410-092902 PMC3858397

[40] Djebali S, Davis CA, Merkel A, Dobin A, Lassmann T, et al (2012) Landscape of transcription in human cells. Nature 489: 101–108. 22955620 10.1038/nature11233 PMC3684276

[41] Birney E, Stamatoyannopoulos JA, Dutta A, Guigo R, Gingeras TR, et al (2007) Identification and analysis of functional elements in 1% of the human genome by the ENCODE pilot project. Nature 447: 799–816. 17571346 10.1038/nature05874 PMC2212820

[42] Sollu C, Pars K, Cornu TI, Thibodeau-Beganny S, Maeder ML, et al (2010) Autonomous zinc-finger nuclease pairs for targeted chromosomal deletion. Nucleic Acids Res 38: 8269–8276. 20716517 10.1093/nar/gkq720 PMC3001086

[43] Lee HJ, Kim E, Kim JS (2010) Targeted chromosomal deletions in human cells using zinc finger nucleases. Genome Res 20: 81–89. 19952142 10.1101/gr.099747.109 PMC2798833

[44] Chen F, Pruett-Miller SM, Huang Y, Gjoka M, Duda K, et al (2011) High-frequency genome editing using ssDNA oligonucleotides with zinc-finger nucleases. Nat Methods 8: 753–755. 21765410 10.1038/nmeth.1653 PMC3617923

[45] Gupta A, Hall VL, Kok FO, Shin M, McNulty JC, et al (2013) Targeted chromosomal deletions and inversions in zebrafish. Genome Res 23: 1008–1017. 23478401 10.1101/gr.154070.112 PMC3668355

[46] Xiao A, Wang Z, Hu Y, Wu Y, Luo Z, et al (2013) Chromosomal deletions and inversions mediated by TALENs and CRISPR/Cas in zebrafish. Nucleic Acids Res 41: e141. 23748566 10.1093/nar/gkt464 PMC3737551

[47] Lim S, Wang Y, Yu X, Huang Y, Featherstone MS, et al (2013) A simple strategy for heritable chromosomal deletions in zebrafish via the combinatorial action of targeting nucleases. Genome Biol 14: R69. 23815890 10.1186/gb-2013-14-7-r69 PMC4054832

[48] Landgraf P, Rusu M, Sheridan R, Sewer A, Iovino N, et al (2007) A mammalian microRNA expression atlas based on small RNA library sequencing. Cell 129: 1401–1414. 17604727 10.1016/j.cell.2007.04.040 PMC2681231

[49] Kim VN, Han J, Siomi MC (2009) Biogenesis of small RNAs in animals. Nat Rev Mol Cell Biol 10: 126–139. 19165215 10.1038/nrm2632

[50] Giraldez AJ, Cinalli RM, Glasner ME, Enright AJ, Thomson JM, et al (2005) MicroRNAs regulate brain morphogenesis in zebrafish. Science 308: 833–838. 15774722 10.1126/science.1109020

[51] Mishima Y, Giraldez AJ, Takeda Y, Fujiwara T, Sakamoto H, et al (2006) Differential regulation of germline mRNAs in soma and germ cells by zebrafish miR-430. Curr Biol 16: 2135–2142. 17084698 10.1016/j.cub.2006.08.086 PMC1764209

[52] Koprunner M, Thisse C, Thisse B, Raz E (2001) A zebrafish nanos-related gene is essential for the development of primordial germ cells. Genes Dev 15: 2877–2885. 11691838 10.1101/gad.212401 PMC312811

