# Supplementary material for: Inheritable and Precise Large Genomic Deletions of Non-Coding RNA Genes in Zebrafish Using TALENs
Source: PLoS One. 2013 Oct 10;8(10):e76387. doi: 10.1371/journal.pone.0076387 (PMC3794983; doi:10.1371/journal.pone.0076387)
Supplement: File S1 — Supporting Figures and Tables. (PDF) [file pone.0076387.s001.pdf]

MiR-1-1: mutations in 64 of 66 sequences (～97.0%)

```
TATGAACAAGAGCAGCTATGGAATGTAAAGAAGTATGTATCCCAGGTGA   WT
TATGAACAAGAGCAGCTATGGAA------------------------->   Δ101
TAT--------------------------GAAGTATGTATCCCAGGTGA   Δ26
TATG---------------------TAAAGAAGTATGTATCCCAGGTGA   Δ21
TATGAACAAGA--------------------actatgttTCCCAGGTGA   Δ20(Δ28,+8)
TATGAACAAGAGCAGCTATG-----------------TATCCCAGGTGA   Δ17
TATGAACAAGAGCAGCTATGGA----------GTATGTATCCCAGGTGA   Δ10          X2
TATGAACAAGAGCAGC----------AAAGAAGTATGTATCCCAGGTGA   Δ10
TATGAACAAGAGCAG----------TAAAGAAGTATGTATCCCAGGTGA   Δ10
TATGAACAAGAGCAGCTATGGA---------AGTATGTATCCCAGGTGA   Δ9           X19
TATGAACAAGAGCAGCTAT---------AGAAGTATGTATCCCAGGTGA   Δ9
TATGAACAAGAGCAGCTAT--------AAGAAGTATGTATCCCAGGTGA   Δ8
TATGAACAAGAGCAGCTATGGA--------gAGTATGTATCCCAGGTGA   Δ8 (Δ9,+1)
TATGAACAAGAGCAGCTATGG-------AGAAGTATGTATCCCAGGTGA   Δ7           X2
TATGAACAAGAGCAGCTATG-------AAGAAGTATGTATCCCAGGTGA   Δ7
TATGAACAAGAGCAGCTAT-------AAAGAAGTATGTATCCCAGGTGA   Δ7           X2
TATGAACAAGAGCAGCTATGGAA------GAAGTATGTATCCCAGGTGA   Δ6           X13
TATGAACAAGAGCAGCTATGGA------tGAAGTATGTATCCCAGGTGA   Δ6 (Δ7,+1)
TATGAACAAGAGCAGCTATGGA------AGAAGTATGAATCCCAGGTGA   Δ6
TATGAACAAGAGCAGCTATG------AAAGAAGTATGTATCCCAGGTGA   Δ6           X2
TATGAACAAGAGCAGCTATGG-----aagaagaTATGTATCCCAGGTGA   Δ5 (Δ12,+7)
TATGAACAAGAGCAGCTATGGAA----AAGAAGTATGTATCCCAGGTGA   Δ4           X2
TATGAACAAGAGCAGCTATGC----TAAAGAAGTATGTATCCCAGGTGA   Δ4
TATGAACAAGAGCAGCTATGGAATGTAA-GAAGTATGTATCCCAGGTGA   Δ1
TATGAACAAGAGCAGCTATGGAAgtatgtAAGAAGTATGTATCCCAGGT   +2 (Δ6,+8)
TATGAACAAGAGCAGCTATGagaagtagaacctAAGAAGTATGTATCCC   +6 (Δ7,+13)
TATGAACAAGAGCAGCTATGGAAagtatgaaaaGTAAAGAAGTATGTAT   +9 (Δ1,+10)
TATGAACAAGAGCAGCTATGGAAgtatgtatcccaggtgaGAAGTATGT   +11(Δ6,+17)
TATGAACAAGAGCAGCTATGGAtgtatgtagtatgtattgtatggatgt   +21(Δ6,+27)
TATGAACAAGAGCAGCTAgtatgaagtatgtataattggagcagctagt   +21(Δ10,+31)
```

Figure S1. Targeted disruption of zebrafish *miR-1-1*. The TALEN binding sites are shown in yellow background. DNA sequence encoding the mature miR-1-1 is underlined, with the seed sequence in bold. The sizes of the insertions (+) or deletions (Δ) and the number of times each mutant allele appearing are shown on the right side of the mutant allele.

A Chromosome 1: 2805894-2807334

CGTGTGACATGTGCTTTGCCATGAGGCCCTCGCTTTTGTAGTATAGAAAG**TCAACCCCATGTACCATC**AATATCTATTG
TATTT**GAATAATTCTACAGGTA**ACAAACTAAC----------------------1.2kb--------------------
TCATT**TATTATCTTAACACTGTAA**TTTTTACACCATGCAA**GGCATGATTAACATTTAGA**CACTTGTTTGCTATGCTGTT
AAGTGTGTTAATGACTTGGGTTTTATTAAACCAACATAATCGGACAAATCAACATAGTCTATCAGCTGCTCTTCAGATT
TATCAAATCCAATTATAAAAATGCAGAGTAAGCCGAGTAATCTAGC

B

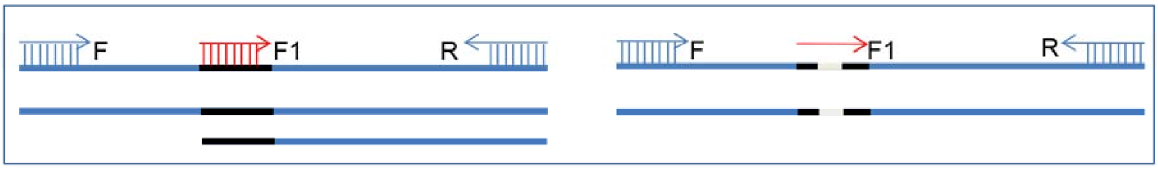

C

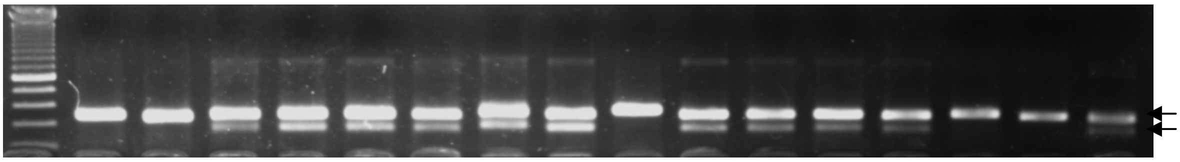

D

TCAACCCCATGTACCATCAATATCTATTGTATTTGAATAATTCTACAGGTA WT
TCAACCCCATGTACCATCAAT-----------TTGAATAATTCTACAGGTA Δ11
TCAACCCCATGTACCATCAATAT---------TTGAATAATTCTACAGGTA Δ9
TCAACCCCATGTACCATCAATAT-----GTATTTGAATAATTCTACAGGTA Δ5 X2
TCAACCCCATGTACCATCAATATCTcaattgtattcaaATTGTATTTGAAT +13 (Δ26, +13)

E

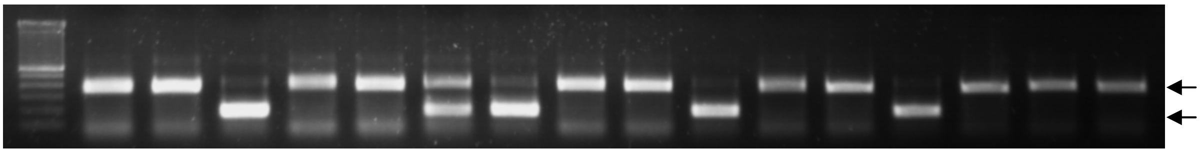

F

TATTATCTTAACACTGTAATTTTTACACCATGCAAGGCATGATTAACATTTAGA WT
TATTATCTTA------------------------gGGCATGATTAACATTTAGA Δ24
TATTATCTTAACACTGTAATT-----------CAAGGCATGATTAACATTTAGA Δ11
TATTATCTTAACACTGTAATTTTTA-------CAAGGCATGATTAACATTTAGA Δ7 X3
TATTATCTTAACACTGTAATTTTT-------GCAAGGCATGATTAACATTTAGA Δ7
TATTATCTTAACACTGTAATTTTT----CATGCAAGGCATGATTAACATTTAGA Δ4
TATTGTCTTAACACTGTAATT----gcaaATGCAAGGCATGATTAACATTTAGA Δ4 (Δ8,+4)
TATTATCTTAACACTGTAATTTTTACAC-ATGCAAGGCATGATTAACATTTAGA Δ4
TATTATCTTAACACTGTAATTTTTACAaattatgaATGCAAGGCATGATTAACA +6 (Δ2,+8)
TATTATCTTAACACTGTAATTTTTtctaaatgttaatCATGCAAGGCATGATTA +9 (Δ4,+13)

Figure S2. Precise large genomic deletion of *miR-17-92* cluster. (**A**) The target genomic region of the zebrafish *miR-17-92* cluster. The TALEN binding sites are shown in color. Primers to detect fragment deletion are underlined. (**B**) Schematic diagram of PCR-based mutation detection. Three primers were designed and used in the PCR reactions. F1 is located at the spacer region. In single clones containing the wild-type sequence, two bands would be amplified. Whereas in clones containing the mutated sequence, one single band would be amplified because changes in the spacer sequence destroy the binding site of the F1 primer. (**C** and **E**) Gel picture of PCR-based detection of T1 (**C**) and T2 (**E**) targeted loci mutations. The two expected bands in the wild-type clones are indicated by arrows. (**D** and **F**) Sequencing results confirming T1 (**D**) and T2 (**F**) targeted loci mutations. Deletions are indicated by dash lines and insertions are

indicated by lowercase letters. The sizes of the insertions (+) or deletions (Δ) and the number of times each mutant allele appearing are shown on the right side of the mutant allele.

A Chrosome 4:27939580-28019747

GTCTGAAATGTCACATTAAAGGATTAATTTCAACAGCATCGCTGTAACAAGTCAATAGGCCATATGCTGTTTGCTGTCATAAGACT
ACAACCATGTTCTACATTTTGTTTTTTATCAACTTTGTGAATCTTGACACATATTGATCATTACTGCTAACGTTATACATATAACA
TGCAGGCT**TCTGAATAGTCACAGTGTC**TGTGTTTAATTAGAACA**ATGTTAAAATACAGATGTA**AATAAATAAGCATGAGCCTTTCA
ATTACGGTTCATGTCCAGTAGAAAAACTAAACAAATGATTTG-------------------79.8kb------------------------
TAGATATATCAGCATTGGTCCCGATAGACTCTGCTAGAGGTTCAATAAATAATGTGAACTACAAGGGTGATAGGGGACAACCCTGT
CTGCAACCTATGCGTACTGGAAA**TCCTTAAGAAGTCAAACC**ATTTGTATTGACCGAG**GCAACAGAATCTGCATAGA**AGATACTTAA
TAACATTAATAAAATTAGAACTTAGTCCAAATTTTTTCAAAGCTAAAAACAAGAAAGGCCACTCTACACAGTAAAAATCCTTTTTG
GCTTCCAGAGATAATATCATTAATGTCTTTTTCTGTGTATGAAAATAATGTGGAGACGTACATTATCCAATCCATGCCTGGATTTC
ACAAAGTCTGCTATTTATAACCTTAGGAAGGATAGATTCCAATCTGCGAGCCA

B

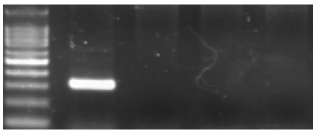

←~320 bp

C miR-430 T1 locus

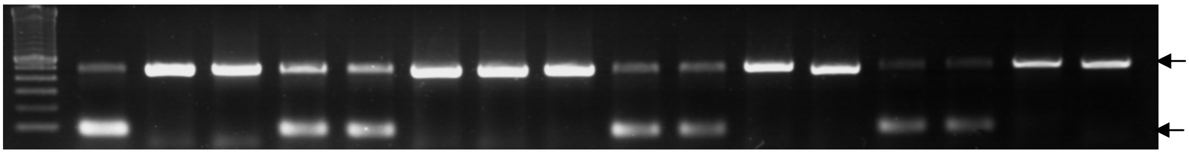

D

TACATCTGTATTTTAACATTGTTCTAATTAAACACAGACACTGTGACTATTCAGA WT
TACATCTGTATTTTAACA----------------CAGACACTGTGACTATTCAGA Δ16
TACATATGTATTTTAACATT------------AACAGACACTGTGACTATTCAGA Δ12
TACATCTGTATTTTAACATTGTTCT-----------GACACTGTGACTATTCAGA Δ11
TACATCTGTATTTTAACATTGTTCTA----------GACACTGTGACTATTCAGA Δ10
TACATCTGTATTTTAACATTGTTT------AACACAGACACTGTGACTATTCAGA Δ6
TACATCTGTATTTTAACATTGTT------AAACACAGACACTGTGACTATTCAGA Δ6
TACATCTGTATTTTAACATTGTTCTgtcaatattttaACAGACACTGTGACTATT +4 (Δ8, +12)
TACATCTGTATTTTAACATTGTTCatctgtattttAACACAGACACTGTGACTAT +5 (Δ6, +11)
TACATCTGTATTTTAACATTgttcattgcaattccatgccaaatgggttcagtct +48(Δ8, +56)
tgcttgttgtacgtgtccttaTAAACACAGACACTGTGACTATTCAGA

E

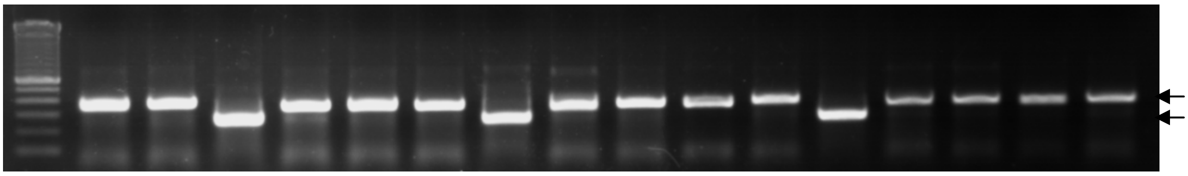

F

TCCTTAAGAAGTCAAACCATTTGTATTGACCGAGGCAACAGAATCTGCATAGA WT
TCCTTAAGAAGTCAAACCA--------------GGCAACAGAATCTGCATAGA Δ14
TCCTTAAGAAGTCAAACC-------------GAGGCAACAGAATCTGCATAGA Δ13
TCCTTAAGAAGTCAAACCATTTG-----------GCAACAGAATCTGCATAGA Δ11
TCCTTAAGAAGTCAAACCAT----------CGAGGCAACAGAATCTGCATAGA Δ10
TCCTTAAGAAGTCAAACCA----------CCGAGGCAACAGAATCTGCATAGA Δ10
TCCTTAAGAAGTCAAACCA---------ACCGAGGCAACAGAATCTGCATAGA Δ9
TCCTTAAGAAGTCAAACCAT--------ctctatGCAACAGAATCTGCATAGA Δ9 (Δ15,+6)
TCCTTAAGAAGTCAAACCATTTGT-------ttGGCAACAGAATCTGCATAGA Δ7 (Δ10,+7)
TCCTTAAGAAGTCAAACCAT-----gttgCCGAGGCAACAGAATCTGCATAGA Δ5 (Δ9,+4)
TCCTTAAGAAGTCAAACCATTTGTAtTTGACCGAGGCAACAGAATCTGCATAG +1
TCCTTAAGAAGTCAAACCATTTGaggcaacacACCGAGGCAACAGAATCTGCA +4 (Δ5,+9)

Figure S3. Precise large genomic deletions of *miR-430* cluster. (**A**) The target genomic region of the zebrafish *miR-430* cluster. The TALEN binding sites are shown in color. Primers to detect fragment deletion are underlined. (**B**) Genomic PCR detection of the *miR-430* cluster deletion. Gel picture showing 40 cycles PCR amplification of genomic DNA isolated from the pooled zebrafish embryos microinjected with the dual TALEN or wildtype control. (**C** and **E**) Gel picture of PCR-based detection of T1(**C**) and T2 (**E**) targeted loci mutations. The two expected bands in the wildtype clones are indicated by arrows. (**D** and **F**) Sequencing results confirming T1 (**D**) and T2 (**F**) targeted loci mutations. Deletions are indicated by dash lines and insertions are indicated by lowercase letters. The sizes of the insertions (+) or deletions (Δ) and the number of times each mutant allele appearing are shown on the right side of the mutant allele.

A Chromsome 14: 48575037-48564858

```
ACACAAACAGGCTAAGATGGCTAGTCTTTCTTGTTTTTTAAGTGTATTTGTGTTCCCATCCCCCTTCCCTTCACATTAGC
AGTCTCGTCAGCACAAATACCAAAAGGAGACGTGCTGGAGGTCTCGTTTACCCCGCACCCACCCGTTACTGTCCAAAGCGAC
CAAGAAACACAGTTTCCTTCAATATATCCCCATTTATTAACATTAAAATCACTTT-------------9.0kb-----------
CAAATCGTGCTCTGGTTCGTATACAAAAGTCAAATCTCTTTTGTAATCTTATACAACATTTATCTATTCTCTGCCTACTTGTT
TTAAGGTAACATTCATGTGAGAACATTTATTGGGGTTTGTTTGAATGTCCATGGGCATTGAAAATAATGGGAAATTGATAA
AGGGTTCGTTTCTCAGAACGATAACCTGTTACAAT
```

B

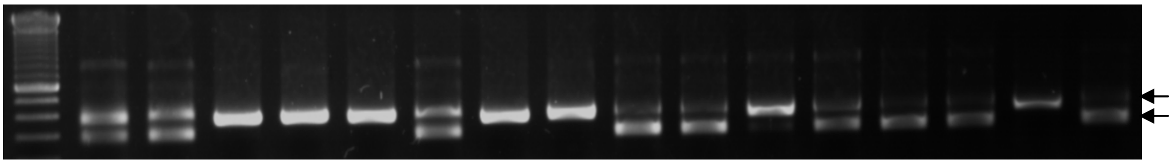

C

```
TAGCAGTCTCGTCAGCACAAATACCAAAAGGAGACGTGCTGGAGGTCTCGTTTA WT
TA-----------------------------------------GTCTCGTTTA Δ42
TAGCAGTCTCGTCAGCACAAATACC-----------TGCTGGAGGTCTCGTTTA Δ11
TAGCAGTCTCGTCAGCACAAATA--------tgtCGTGCTGGAGGTCTCGTTTA Δ8
TAGCAGTCTCGTCAGCACAAATACCAA--GGAGACGTGCTGGAGGTCTCGTTTA Δ2
TAGCAGTCTCGTCAGCACAAATACCgtgcaataaACGTGCTGGAGGTCTCGTTT +1(Δ8,+9)
```

D

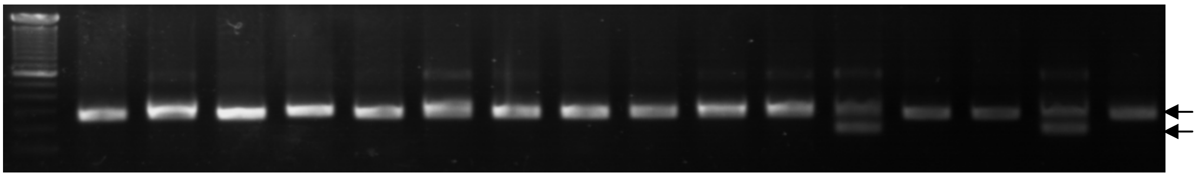

E

```
TATCTATTCTCTGCCTACTTGTTTTAAGGTAACATTCATGTGAGAACATTTA WT
TATCTATTCTCTGCCTACTTGT--------AACATTCATGTGAGAACATTTA Δ8 X2
TATCTATTCTCTGCCTACTTGTTT--------CATTCATGTGAGAACATTTA Δ8 X2
TATCTATTCTCTGCCTACTTGTTT-----TAACATTCATGTGAGAACATTTA Δ5
TATCTATTCTCTGCCaacatgtaatgtactctGGTAACATTCATGTGAGAAC +5 (Δ12,+17)
TATCTATTCTCTGCCTACTTGTTTcatgtttctctgcctacttCATTCATGT +11(Δ8,+19)
```

Figure S4. Precise large genomic deletions of *malat1*. (**A**) The target genomic region of the zebrafish *malat1*. The TALEN binding sites are shown in color. Primers to detect fragment deletion are underlined. (**B** and **D**) Gel picture of PCR-based detection of T1 (**B**) and T2 (**D**) targeted loci mutations. The two expected bands in the wildtype clones are indicated by arrows. (**C** and **E**) Sequencing results confirming T1 (**C**) and T2 (**E**) targeted loci mutations. Deletions are indicated by dash lines and insertions are indicated by lowercase letters. The sizes of the insertions (+) or deletions (Δ) and the number of times each mutant allele appearing are shown on the right side of the mutant allele.

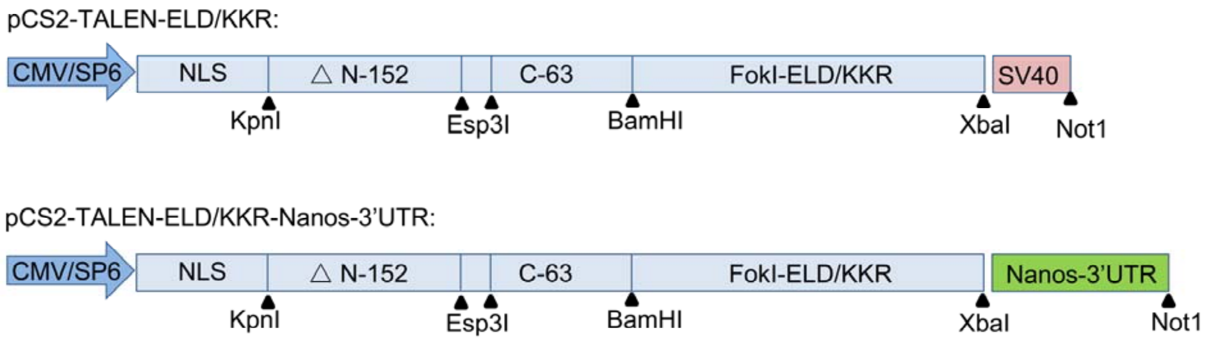

Figure S5. Incorporation of nanos-3'UTR into the pCS2-TALEN-ELD/KKR vector. Black triangles, endonuclease recognition sites; CMV/SP6, CMV/SP6 promoters; NLS, nuclear localization signal; SV40, SV40 polyadenylation signal sequence; △N152-Esp3I-C63, TALE scaffold with truncation of the N-terminal 152-aa and retention of C-terminal 63-aa, containing two Esp3I sites where the TALE repeats could be inserted.

A

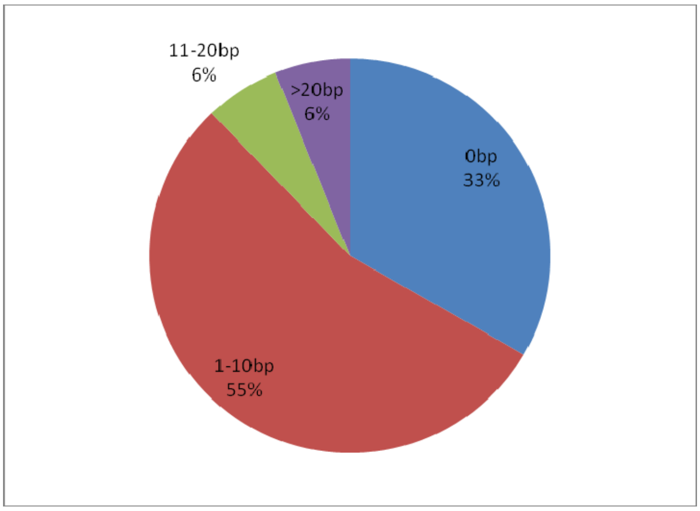

B

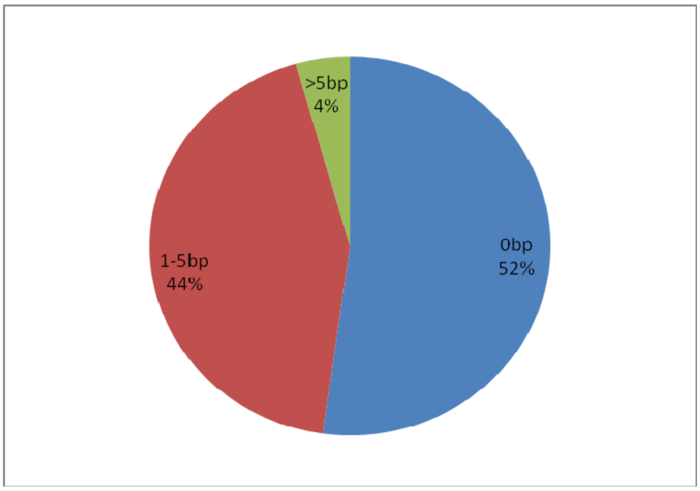

Figure S6. Proportion of the different ranges of alien nucleotides retained after large genomic deletions in somatic cells (total=33) (**A**) and germ cells (total=23) (**B**). Data were from the sequencing results of the *miR-17-92*, *miR-430* and *malat1*.

Table S1 TALEN injection records

TALEN targets	mRNA amount	Total embryos injected	Day1 #	Day2 #
MiR-1-1	200pg	33	29(0)	28(0)
MiR-1-2	200pg	41	37(5)	32(2)
MiR-430T1T2	100pg	174	156(14)	136(8)
MiR-430T1T2-nos1-3'UTR	100pg	152	146(6)	126(8)
Malat1T1T2	100pg	140	121(10)	112(9)
MiR-17-92T1T2	100pg	87	78(7)	68(4)

The total number of surviving embryos are shown, with the number of deformed embryos in brackets.

Table S2 Primers used in the present study

Primer name	Primer sequence (5′-3′)	Purpose
pCR8_F1<br>pCR8_R1	TTGATGCCTGGCAGTTCCCT<br>CGAACCGAACAGGCTTATGT	Amplification and sequencing of the assembled TALEs in the array plasmids.
NTalF<br>CTalR	GATGACAAGGGTACCGTG<br>CTAGTTGGGATCCGGCAAC	Amplification and sequencing of the assembled TALENs.
MiR-1-1F<br>MiR-1-1R	GTCTAAATGCTCATATCTGAGG<br>CATCAGGCCTGCGCATCACAC	*MiR-1-1* genomic fragment amplification.
MiR-1-2F<br>MiR-1-2R	CTAATCCACTGCATTGTGCAG<br>GTGGACTGCTGGTGAAGTTACTG	*MiR-1-2* genomic fragment amplification.
MiR-430F1<br>MiR-430R1<br>MiR-430M1<br>MiR-430F2<br>MiR-430R2<br>MiR-430M2<br>MiR-430F3<br>MiR-430R3	CACATATTGATCATTACTGCTAAC<br>GTCAGGTAGGTCTTACGCACAC<br>ACATTGTTCTAATTAAACACAG<br>CTGACAGCAACGGGAACAGATG<br>CTCGCAGATTGGAATCTATCCTTC<br>CAAACCATTTGTATTGACCGAG<br>CAGCATCGCTGTAACAAGTC<br>GTATAACGTTAGCAGTAATG	MiR-430T1 and miR-430T2 genomic loci amplification.<br>PCR-based mutation detection.<br>MiR-430F1 + MiR-430R2 for detection of *miR-430* cluster deletion.<br>MiR-430F3 + MiR-430R3 for amplification of reference fragment.
Malat1F1<br>Malat1R1<br>Malat1M1<br>Malat1F2<br>Malat1R2<br>Malat1M2<br>Malat1F3<br>Malat1R3	CAAACAGGCTAAGATGGCTAGTC<br>AAGTTGTACAAACGCAGTACAG<br>CACAAATACCAAAAGGAGACG<br>AGGTGCATTTAGCTGATGTAGAG<br>GTAACAGGTTATCGTTCTGAG<br>CATGAATGTTACCTTAAAACAAG<br>GACTCACAACATCATGACGACAG<br>TCTACTGACCGGTACAAACCTG	Malat1T1 and Malat1T2 genomic loci amplification. PCR-based mutation detection. Malat1F1 + Malat1R2 for detection of cluster deletion; for amplification of reference fragment. Malat1F3 + Malat1R3 for amplification of reference fragment.
MiR-17-92F1<br>MiR-17-92R1<br>MiR-17-92M1<br>MiR-17-92F2<br>MiR-17-92R2<br>MiR-17-92M2<br>MiR-17-92F	GTGACATGTGCTTTGCCATGAG<br>GTTGGGTGTCTTGCCGAAGGATG<br>CATCAATATCTATTGTATTTG<br>GTAAAGGATTGTGGAGATTGTACC<br>GACAAAACTTCAGCAGTGAACACAG<br>GTAATTTTTACACCATGCAAG<br>GTACACATGCTTAATGCAGAGG	MiR-17-92T1 and miR-17-92T2 genomic loci amplification. PCR-based mutation detection. MiR-17-92F1 + MiR-17-92R2 for detection of cluster deletion; Mir-17-92F + Mir-17-92R2 for amplification of reference fragment.

Table S3 Mutation frequencies of the T1 and T2 targeted loci

TALEN name	Mutant alleles /total alleles	Mutation frequency (%)	TALEN name	Mutant alleles /total alleles	Mutation frequency (%)
MiR-430T1	14/32	43.8	MiR-430T2	27/32	84.4
MiR-430T1	17/32	53.1	MiR-430T2	26/32	81.3
MiR-430T1	12/32	37.5	MiR-430T2	20/32	62.5
Malat1T1	16/32	50	Malat1T2	26/32	81.3
Malat1T1	16/32	50	Malat1T2	29/32	90.6
Malat1T1	13/32	40.6	Malat1T2	29/32	90.6
MiR-17-92T1	8/32	25	MiR-17-92T2	24/31	77.4
MiR-17-92T1	3/27	11.1	MiR-17-92T2	23/31	74.2
MiR-17-92T1	4/32	12.5	MiR-17-92T2	23/32	71.9
